# Processing Polymer Blends of Mater-Bi^®^ and Poly-L-(Lactic Acid) for Blown Film Application with Enhanced Mechanical Strength

**DOI:** 10.3390/polym15010153

**Published:** 2022-12-29

**Authors:** Samar Bouzidi, Emna Ben ayed, Quim Tarrés, Marc Delgado-Aguilar, Sami Boufi

**Affiliations:** 1 University of Sfax, LMSE, Faculty of Science, BP 802, Sfax 3018, Tunisia; 2 LEPAMAP-PRODIS Research Group, University of Girona, Maria Aurèlia Capmany 61, 17003 Girona, Spain

**Keywords:** blends, Mater-Bi, BLOW film, extrusion, biodegradable

## Abstract

Mater-Bi^®^ is one of the most commercialized starch-based blends used in biodegradable flexible packaging. However, the high ductility and low stiffness of Mater-Bi^®^ might limit its application and developing a solution to tailor the stiffness and mechanical strength is highly desirable. In the present work, blends based on Mater-Bi^®^ and poly-L-(lactic acid) (PLLA) at a different ratio from 70/30 to 50/50 wt% were prepared via melt-extrusion and the effect of the PLLA content and Joncryl ADR^®^ as a reactive compatibilizing agent, on the mechanical properties, melts rheology, morphology and disintegration aptitude were investigated. The inclusion of PLLA in Mater-Bi^®^ has a marked beneficial effect on the tensile strength and stiffness of the blend while maintaining acceptable ductility. The addition of the reactive compatibilizing agent contributed to improving the strength and elongation at the break of the blend. The melt rheology of the blend was also affected by the ratio of the two components, mostly when the Joncryl ADR^®^ was present. The disintegration by biodegradation of the blend was preserved in the presence of PLLA, and it takes less than 30 days for the films to completely decompose and disintegrate under controlled composting conditions. Interestingly, a thin film from Mater-Bi^®^/PLLA 60/40 was successfully prepared by blown film extrusion, demonstrating a good balance between stretchability (elongation at break exceeding 100%) and stiffness (1.8 GPa). This work opened to broadening the use of starch-based biodegradable plastic toward more demanding applications such as mulching films.

## 1. Introduction

Biodegradable polymers are those that are susceptible to break down by microorganisms and decompose into environmentally acceptable substances such as water, carbon dioxide (CO_2_) (under aerobic environments), methane (CH_4_) (under anaerobic conditions), and biomass [1]. Although the term ”biodegradable polymers” sounds tempting, especially after decades of efforts to solve the major plastic pollution crisis, it was found that not all biodegradable polymers, whether they are derived from renewable resources or synthetic biodegradable ones, are regarded as fully compostable, even though the two terms were often used interchangeably causing a lot of confusions [2]. Poly-L-(lactic acid) (PLLA), for example, is considered a potentially sustainable alternative to petroleum-based polymers in a broad range of applications, including biomedical [3], food packaging [4], and commodity products [5]. This increasing interest in PLLA is ascribed to its bio-based origin, biocompatibility, good biodegradability, and excellent mechanical properties [3,4]. Unfortunately, despite all these attributes, the inherent brittleness, high stiffness, and ductility narrow its application window in comparison to commodity polyolefins, which challenge its large-scale manufacturing into stretchable films that can be attained via film-blowing or cast film extrusion process [6]. Compared to polyolefins, PLLA has weaker melt strength, and therefore, the formation of a stable bubble during extrusion blowing is more difficult. The high stiffness of PLLA results in the formation of wrinkles in the film during the collapsing of the bubble in the nips rolls PLLA [6]. Another major drawback of PLLA is its compostability characteristics under different environmental conditions. PLLA can take decades to decompose in seawater, soil, landfills, and at-home composting conditions (<37 °C) [7], while it decomposes within 180 days in industrial facilities (>55 °C) [8]. One of the alternatives to alleviate the shortcomings of PLLA is blending it with soft biodegradable polymers to enhance the ductility and toughness of PLLA. Under this approach, a lot of combinations of PLLA with ductile polyester were reported, and the effects on the properties of the blends in terms of their mechanical, thermal, and biodegradability were investigated [5,9,10]. For instance, it was shown that the inclusion of PHB or PBAT in PLLA enhanced the biodegradation of the blend under aerobic conditions [11]. The inclusion of thermoplastic starch (TPS) on binary PLLA/PBAT blend also proved to be efficient in accelerating the disintegration process in ambient conditions (home composting) [9] of PLLA/PBAT blend that was compounded via a co-rotating reactive extruder.

Mater-Bi^®^ is a commercial biodegradable polymer based on a blend of polybutylene adipate terephthalate (PBAT) and TPS, capable of being processed by extrusion-blowing or injection molding to produce wide biodegradable materials, including carrier bags, agricultural mulch film, nets for fresh fruit and vegetables, plates, cutlery, cups, and spoons. However, the high ductility and low stiffness of Mater-Bi^®^ are shortcomings that limit the application of Mater-Bi^®^ [12,13]. Blending Mater-Bi^®^ with PLLA may be a useful strategy to design biodegradable material with a compromise in their thermomechanical, chemical and physical characteristics suitable for thin film processing via extrusion-blowing [12]. Only a limited number of works have been reported concerning Mater-Bi^®^/PLLA blends. Mistretta et al. [14] demonstrated the effectiveness of blending Mater-Bi^®^ and PLLA reinforced by two types of nanoclays via a twin-screw extrusion process to produce blown packaging films with mechanical performances comparable to petroleum-based polymers exploited in packaging industries. Scaffaro et al. [15] investigated the rheological, mechanical, and optical properties of 100% green and biodegradable films based on PLLA/Mater-Bi^®^ filled with a leave extract (Posidonia Oceanica).

Up until very recently, Serra-Parareda et al. [12] reported a study in which they compounded PLLA with Mater-Bi^®^ and bleached Kraft hardwood fibers by melt processing to alter the mechanical properties of the fabricated composites and its increased compostability thanks to the presence of the Mater-Bi^®^ polymer in the formulation. On the other hand, the use of Joncryl ADR^®^ as a coupling agent in PLA blends and compounds has been reported in the literature [16]. Joncryl ADR^®^ is a multifunctional polymer chain extender epoxy; stated otherwise, it has a high compatibilizing functionality as a structural material. This reagent intertwines with the different polymer chains and creates a better bond between them, resulting in improved physical, mechanical, and thermal properties. Therefore, this additive improves the interface between the polymeric materials in the matrix. This fact can be seen experimentally with a drastic change in the morphology of the resulting material. The mechanical properties are also affected by this morphological change, with an increase of the elongation under stress, i.e., providing a higher malleability. Conversely, the Young’s modulus decreases, reducing the fragility and stiffness of the resulting composite.

In this present study, aiming to enhance the mechanical performance of Mater-Bi^®^ films, PLLA was added with different contents to the Mater-Bi^®^, which were subsequently compounded using the melt-blending method in a twin screw extruder. An epoxy-based compatibilizer was included to ensure better interfacial compatibility and enhanced physical properties of the films. The thermomechanical, morphological, thermal, and rheological properties of the blends were investigated. Moreover, the disintegration of the blended films under industrial compositing conditions was assessed. Finally, the best Mater-Bi^®^/PLLA composition was tested to produce thin films by extrusion-blowing using a pilot-scale extrusion-blowing machine.

## 2. Materials and Methods

### 2.1. Materials

The main component of the studied blends is a thermoplastic starch-based polymer named Mater-Bi^®^_YI0140/C (MB) that was purchased from Novamont (Novara, Italy). Poly (Lactic acid) (PLLA) pellets were supplied by Natureworks (Blair, Nebraska) under the trade name Ingeo^®^ biopolymer 3D870. The compatibilizer compounded with the two biopolymers is a commercial multifunctional epoxide type that goes by the name Joncryl-ADR^®^ 4468 (JCL) (From BASF, Ludwigshafen, Germany). Table 1 presents the more detailed characteristics of the polymers involved in this study.

### 2.2. Blend Preparation

Mater-Bi^®^ and PLLA pellets were dried for 4 h at 70 °C prior to processing. Afterward, the pellets of the two biopolymers and the compatibilizer Joncryl-ADR^®^ were weight and compounded according to the summarized composition in Table 2: using a twin screw extruder (Letsritz 18 MAXX) with a screw diameter of 18.5 mm and screw length of 32D. The speed of the screw was 50 rpm with a temperature profile ranging from 155–165 °C from the hopper to the die. For the film-blowing process, each extrudate obtained from the melt was quenched in a cold water bath and granulated by an automatic knife cutter, then kept in the oven overnight at 70 °C.

The composition with 60/40 for the incorporation of Joncryl-ADR^®^ was used as an intermediate value in the mechanical properties between the matrix and the blends studied. It is considered a representative composition of the behavior of the blends with the Joncryl-ADR^®^ compatibilizer. Only 2% of the compatibilizing agent Jocryl-ADR^®^ was used to avoid an influence of the compatibilizing agent on the mechanical properties by its presence and not by the interaction in the blend. It is known that this type of coupling agent must be used in low percentages for their effectiveness [16].

### 2.3. Films Blowing

The blown films were prepared after drying the blends granules for 24 h at 70 °C; then, the films were fed to the hopper of the single screw film blowing pilot extrusion (Screw 20 mm, L/D:28:1) (SCM20, Lianjiang Machienry, Zhangjiagang, China). The blown extrusion process was carried out at different temperature zones that were set from 195 °C to 185 °C with a screw speed of 250 rpm. The investigated parameters in this study are the blow-up ratio (BUR), the draw-down ratio (DDR), and the forming ratio (FR). The BUR is defined by Equation (1):

(1) $$\mathrm{BUR}=\frac{\mathrm{diameter}\;\mathrm{of}\;\mathrm{the}\;\mathrm{bubble}\;\left(\mathrm{Db}\right)}{\mathrm{Diameter}\;\mathrm{of}\;\mathrm{the}\;\mathrm{Die}\;\left(\mathrm{Dd}\right)},$$

Since it is difficult to measure the diameter of the bubble unless it is knocked down, a more practical formula can be used to calculate the BUR, Equation (2):

(2) $$\mathrm{BUR}=\frac{\mathrm{Lay}\;\mathrm{flat}\;\mathrm{width}\;\mathrm{of}\;\mathrm{the}\;\mathrm{bubble}\;\times \;0.637}{\mathrm{Diameter}\;\mathrm{of}\;\mathrm{the}\;\mathrm{Die}\;\left(\mathrm{Dd}\right)},$$

The DDR is defined as the reduction of the final thickness in the melt after blowing and is measured according to Equation (3).

(3) $$\mathrm{DDR}=\frac{\mathrm{Width}\;\mathrm{of}\;\mathrm{the}\;\mathrm{die}\;\mathrm{gap}\;}{\mathrm{film}\;\mathrm{thickness}\;\times \;\mathrm{BUR}}$$

The blowing and processing parameters are presented in Table 3.

### 2.4. Differential Scanning Calorimetry (DSC)

About 2–3 mg of Mater-Bi^®^/PLLA films were weighed and sealed in aluminum pans. Initially, the pans were placed in an autosampler of a DSC analyzer (Perkin-Elmer Pyris Diamond DSC) and then tested under nitrogen flow. The protocol of heating/cooling was as follows: First heating scan from −20 °C to 200 °C and then cooled down to 20 °C and finally, a second heating scan from −20 °C to 200 °C at 10 °C·min^−1^.

Isothermal crystallization was performed as follows. First, the samples were heated to 200 °C for 5 min to erase the thermal history, then quenched at a rate of 50 °C·min^−1^, reheated to the desired isothermal temperature (Tc = 110, 115, 120, and 125 °C) at a rate of 10 °C·min^−1^ and held for 30 min to allow complete crystallization from the quiescent melt. The degree of crystallinity, *Xc*, was calculated using Equation (4):

(4) $$X_{c}\left(\%\right)=\frac{100\cdot \;(\Delta H_{m}-\Delta H_{cc})}{\Delta H_{m}^{0}\cdot w_{PLLA}},$$

where Δ*H_m_* and Δ*H_cc_* are the melting enthalpies and the cold crystallization, respectively, $\Delta H_{m}^{0}$ is the theoretical melting enthalpy of 100% crystalline PLLA (93.1 J g^−1^) and $w_{PLLA}$ is the weight fraction of *PLLA* in the composite.

For the Avrami analysis, the relative crystallinity conversion $\chi_{c}$, was calculated using Equation (5):

(5) $$\chi_{c}=\frac{\Delta H\left(t\right)}{\Delta H_{total}},$$

The isotherms were analyzed in Origin^®^ using a special plugin developed by Lorenzo et al. for Avrami and the Lauritzen–Hoffman studies [17]. The mass fraction of crystalline material, $W_{c}$, is proportional to $\chi_{c}$. The volume fraction of converted material (*V_c_*) is computed using Equation (3), where the densities of amorphous and crystalline PLLA are assumed to be $\rho_{a}$ = 1.25 g/cc and $\rho_{c}$ = 1.359 g/cc, respectively. The Avrami plots were then obtained using Equation (6).

(6) $$V_{c}=\frac{W_{c}}{Wc+\left(\frac{\rho_{c}}{\rho_{a}}\right)\cdot \;\left(1-W_{c}\right)}\;1-V_{c}\left(t\right)=e^{\left(-kt^{n}\right)}\;Log\left(-Ln\left(1-V_{c}\left(t\right)\right)\right)=Log\left(k\right)+nLog\left(t\right),$$

The crystallization half-time (*t*_1/2_) was calculated using Equation (7):

(7) $$t_{1/2}={\left(\frac{ln2}{k}\right)}^{1/n},$$

### 2.5. Morphological Characterization

The morphology of neat and compatibilized Mater-Bi^®^/PLLA Films was explored using a Zeiss DSM 960A SEM microscope (Carl Zeiss Iberia, Madrid, Spain). For correct and high-quality observations, the surfaces of the samples were coated with gold.

### 2.6. Rheological Properties in a Molten State

The rheological properties of the Mater-Bi^®^/PLLA blends were investigated with a controlled rate dynamic-mechanical rheometer (Pro+Kinexus from Malvern) using a plate–plate geometry with a diameter of 25 mm and a gap inferior to 2 mm. the storage modulus, the loss modulus and the complex viscosity of the blended films were measured as a function of frequency within the range of 0.01–100 rad/s at 165 °C. Before every measurement, the linear viscoelastic region was determined by an amplitude sweep in the deformation range of 0.1100% at frequencies of 1 and 50 rad/s. Subsequently, the deformation during the frequency sweeps was set to be within the linear viscoelastic region.

### 2.7. Dynamic Mechanical Analysis (DMA)

The storage modulus E’, Loss Modulus E”, and Tan δ were measured in tension mode using a PYRIS Diamond DMA (Perkin-Elmer, Waltham, MA, USA). Measurements were performed on the blended films (dimensions: 20 mm-Long, 5 mm-Width, and 0.1–0.3 mm-thick) at 1 Hz with a heating rate of 2 °C·min^−1^ at an amplitude of 10 μm over the temperature range of −80 to 120 °C.

### 2.8. Tensile Tests

Tensile tests were determined using an Instron tensile test machine under temperature with a crosshead speed of 10 mm/min. The specimen dimension was 40 mm × 8 mm × 2 mm, denoting the length, width, and thickness. Ten specimens were tested for each composition. As for the blown film, the same parameters were applied in the extrusion direction (ED) and transverse direction (TD).

## 3. Results and Discussion

### 3.1. Tensile Tests

**Figure 1:**
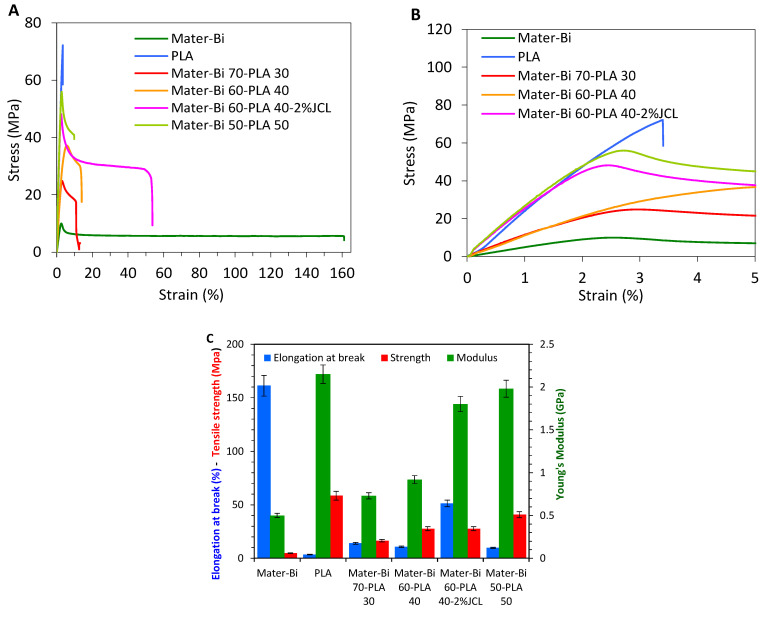
(**A**,**B**) Tensile stress–strain curves of different compositions of Mater-Bi^®^/PLLA blends; (**C**) tensile strength, Young’s modulus, and elongation at break for Mater-Bi^®^/PLLA blends.

The evolution of the mechanical properties of the Mater-Bi^®^/PLLA blends was studied by tensile tests and DMA measurements performed on thin film produced by TSE. The stress–strain curves of different compositions are shown in Figure 1A, from which the tensile strength, tensile modulus, % elongation at break, and toughness were extracted. From the tensile tests, it was clear that neat PLLA possessed the highest tensile strength (≈70 MPa) and the lowest elongation at the break, with only 3.4%. This indicates that PLLA is indeed hard and brittle polyester contrary to neat Mater-Bi^®,^ which presented the features of a ductile plastic with a high elongation at break (161%) but suffers from a low tensile strength near 10.8 MPa. The inclusion of PLLA onto Mater-Bi^®^ at different contents that ranges from 30 to 50 wt% strongly enhanced the strength and stiffness of the blend. Indeed, as shown in Figure 1C, the increment in the ultimate tensile strength/modulus (UTS/E) concerning the neat Mater-Bi^®^ reached about 141/46; 237/78; 410/295%, in the presence of 30, 40, and 50 wt% of PLLA, respectively. However, a decline in the elongation at the break was observed to be higher as the content in the PLLA increased. This effect is expected given the heterophase microstructure of the Mater Bi^®^/PLLA blends, resulting in the premature breaking of the material in the case of poor interfacial adhesion between the continuous and dispersed phase [18] because the dispersed particles acted as stress concentrator from which cracks were initiated and propagated easily in the absence of effective interfacial adhesion between the two phases. Therefore, to attenuate the poor interfacial adhesion and enhance the compatibility between the two immiscible phases, compatibilizers were proven to be very efficient in improving the mechanical properties of binary blends through chemical, covalent, or hydrogen bonds at the interface.

It is well documented that the tensile strength is the most affected tensile parameters after incorporating compatibilizers. An improved interfacial adhesion force leads to a better stress transfer at the interface [19] and the elongation at break [3]. For this purpose, the formulation (Mater-Bi^®^/PLLA 60/40) that exhibited the most intermediate behavior compared to two homopolymers was chosen and compatibilized with Joncryl-ADR^®^ (JCL). The inclusion of 2% JCL in the Mater-Bi^®^/PLLA blend 60/40 led to an obvious improvement in the ultimate tensile strength and attenuated the premature breaking of the blend. In the presence of JCL, the increment in the UTS/E was 357/260 against 237/78 for the uncompatibilized blend. At the same time, the elongation at break reached a value of 51.3% for the compatibilized system vs. 10.8% for the neat blend. These findings could be explained by the fact that the reactive epoxy groups of Joncryl-ADR^®^ during the melt processing promoted chain entanglements and subsequently led to improved toughness and better adhesion between the two phases [19,20]. The addition of Joncryl-ADR^®^ as a chain extender results in a reaction between the carboxylic acid groups of the matrices and the Joncryl-ADR. This causes a structural modification during mixing, which results in the formation of PLLA as ellipsoids, as deformed droplets with low aspect ratio, and possibly acting as stress concentrators in the MaterBi matrix [16]. Further confirmations will be revealed by morphological analysis.

### 3.2. SEM Analysis

**Figure 2:**
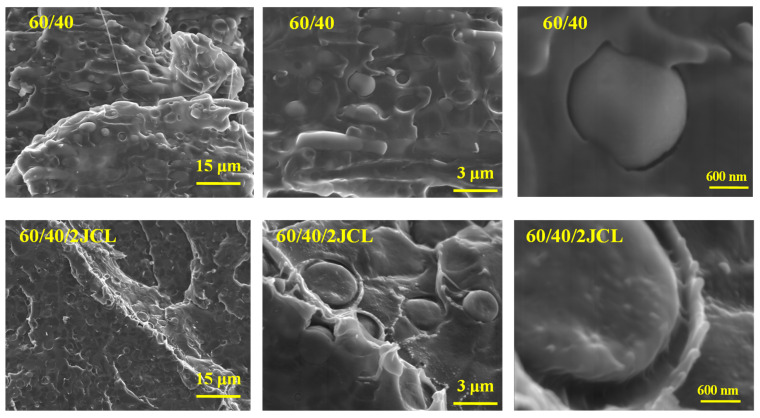
SEM images of the cryogenic fractured surfaces of the 60MB/40, 60MB/40PLLA/2JCL.

The morphology of the polymer blends is a very important characteristic that leads to a better understanding of the mechanical and rheological properties of the blends. The cryo-fractured surface of the films presented in Figure 2 illustrates a classic matrix-dispersed phase morphology in which the Mater-Bi^®^ is the continuous phase, whereas PLLA is the dispersed one in the blend Mater-Bi70-PLLA30 (Figure S1). The same tendency was observed for the blends with Mater-Bi60-PLLA40, while for the composition with an equal part of MB and PLLA (Mater-Bi50-PLLA50), it was not evident to discern between the two phases. The heterogeneous morphology for Mater-Bi/PLLA blends is expected, given the dissimilarity in the chemical structure of Mater-Bi and PLLA, favoring thermodynamic incompatibility between the two phases [21]. To overcome the immiscibility of the two biopolymers, Joncryl-ADR^®^ (JCL) was added to the blend Mater-Bi^®^/PLLA 60/40 at a level of 2 wt%. The choice of JCL was based on literature data demonstrating the efficiency of this reactive coupling agent in improving the compatibility between PLLA and PBAT blends [22,23,24]. In our study, the effect of Joncryl-ADR^®^ was already noticeable in the tensile tests and rheological measurements.

From the SEM micrographs (Figure 2), the decrease in the average size of PLLA particles was insignificant after adding the Joncryl; the structure shown in the same image at a magnitude of 3 μm reveals a more refined morphology and better dispersion of the minor phase. This could be explained by forming chemical bonds between the Mater-Bi^®^/PLLA and the Joncryl functions. It was highlighted in the literature that during the reactive blending process, the epoxy groups of Joncryl react with both groups of the polyesters resulting in extension and a branching reaction. Subsequently, these formed copolymer chains act as a bridge to improve the compatibility of the polymers [22,23,24,25].

### 3.3. Dynamic Mechanical Analysis (DMA)

**Figure 3:**
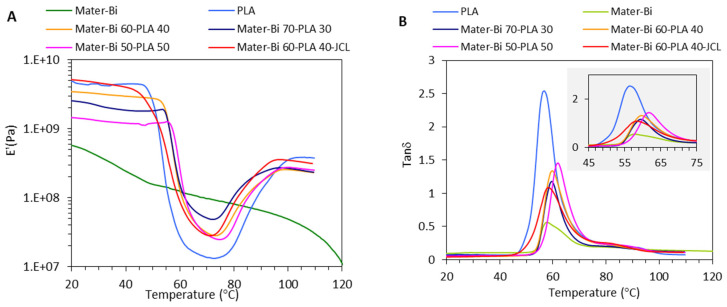
Storage Modulus E’ (**A**), Tan δ (**B**) versus Temperature of Mater-Bi^®^/PLLA blends.

The mechanical properties were also investigated by DMA to probe the stiffness evolution assessed by the storage modulus (E’) as a function of temperature. Figure 3 displays the evolution of E’ as a function of increasing temperature for neat materials, Mater-Bi^®^, and PLLA and their corresponding blends with different content in PLLA. The neat Mater-Bi displays the behavior of a highly ductile polymer with a glassy plateau below −40 °C (Figure S2), followed by a first abrupt fall in E’ by about one order of magnitude, followed by a second transition, presumably due to the plasticized starch phase around 30 °C, and finally E’ sharply decreased over 110 °C due to the melting of the polyester phase (presumably PBAT). These transitions are accompanied by a maximum in tan δ. At a temperature of 20–30 °C, the value of E’ is in the range of 0.3–0.6 GPa, which agrees with tensile test data. Neat PLLA is characterized by a high E’ in the range of 2 GPa maintained up to 50 °C, followed by a sharp decrease in E’ by more than orders of magnitude related to its glass transition. Between 75 to 95 °C, E’ increased again due to the cold crystallization process that induces the rearrangement of the amorphous PLLA chains into crystalline domains with high stiffness. The magnitude of the jump in E’ is determined by the crystalline degree of the PLLA phase; the higher the crystalline degree of PLLA, the lower the increment in E’ at the cold crystallization process. This, in turn, will also affect the amplitude of the decrease of the rigidity of PLLA over the Tg.

The evolution of tan δ vs. temperature revealed a maximum of around 60 °C for PLA and Mater-Bi/PLA blends associated with the Tg of PLLA. The position of this relaxation was slightly different according to the composition (Figure 3B). The lowest Tg was observed for neat PLLA around 56 °C, while the Tg was shifted to 60 and 62 °C for Mater-Bi60-PLA40 and Mater-Bi60PLA40-JCL, respectively. We attribute this evolution to the difference in the crystalline degree between neat PLA (around 6%) and Mater-Bi60-PLA40 and Mater-Bi600PLA40-JCL (around 54%). Since the crystalline phase induces a restriction in the segmental mobility of the amorphous polymer chains in the vicinity of the crystalline lamellar of PLA, the marked increase in the crystalline degree of PLLA in the blend will inevitably result in a shift of the Tg to a higher temperature.

With the inclusion of PLLA, the E’ plateau between 20–50 °C was shifted to a higher value, indicating an enhancement in the stiffness degree of the blend, which is in agreement with tensile-test data at 25 °C. The E’ value increased from 0.77 GPa for neat Mater Bi^®^ to about 2.4 GP, 3.3 GP, and 5 GPa at PLLA content of 30, 40 and 50 wt% in the Mater-Bi^®^/PLLA blends, respectively. In the presence of the coupling agent JCL, the Mater-Bi^®^/PLLA blend at 40% PLLA content displayed a plateau of around 5 GPa, higher than that processed in the absence of JCL. This emphasizes the beneficial role of JCL for the gain in the rigidity of the Mater Bi^®^/PLLA blends, resulting from the improvement of the interfacial adhesion between the PLLA hard phase and the ductile Mater-Bi matrix. As suggested for the PLLA/PBAT blends, the enhancement in the interfacial adhesion between the two-phase results of the chemical through the terminal epoxy groups of JCL with the functional groups (hydroxyl and carboxylic groups) of the Mater-Bi^®^ and PLLA phases, thus generating branched polymers with high affinities to PLLA and the ductile polyester [20]. However, as shown in Figure 3A, the rigidity of the blend decreased again at the onset of the glass transition of the PLLA, with a magnitude that is proportional to the content of PLLA for the uncoupled blends. Then, the E’ increased again for neat PLLA once the cold crystallization of PLLA was activated. In the presence of JCL, both the decrease and regaining of the rigidity after softening of the PLLA and cold crystallization of the PLLA phase were attenuated in comparison with the uncoupled blend at the same PLLA content of 40%.

**Figure 4:**
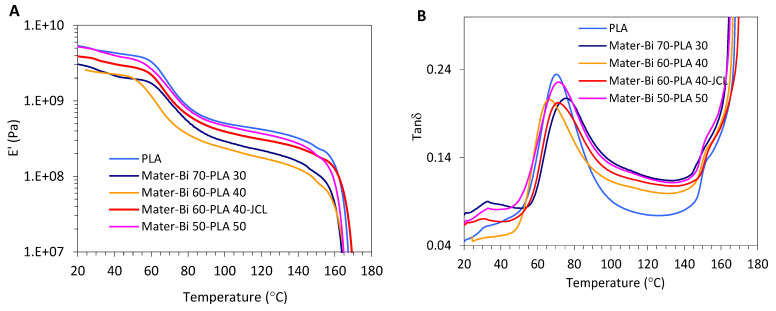
Plot evolution of the storage Modulus E’ (**A**), Tan δ (**B**) versus Temperature of Mater-Bi^®^/PLLA blends after the annealing treatment at 80 °C for 15 min.

Figure 4A,B presents the results of the storage modulus (E’) and tan δ as a function of the temperature of annealed Mater-Bi/PLLA films after annealing for 15 min at a temperature of 80 °C. The shape of the modulus E’ curves for all the formations changed, taking the form of a drop, rise, and drop pattern, which corresponded to the recrystallization process. Usually, the recrystallization process improves the stiffening of the macromolecular chains and, thus, the increment of E’. At a low-temperature range (T < Tg), E’ almost stayed invariable for all the formations and the parent biopolymers. At higher temperatures, E’ increased sharply after annealing and no cold crystallization during the heating process was detected. These results are expected and supported by DSC (Section 3.4) analysis that proved the strong acceleration of the crystallization rate of PLLA because of the presence of Mater-Bi that imparts the formation of crystallites, which in turn forms a dense crystal network and consequently elevate the modulus E’ [18,26,27].

### 3.4. Thermal Properties

**Figure 5:**
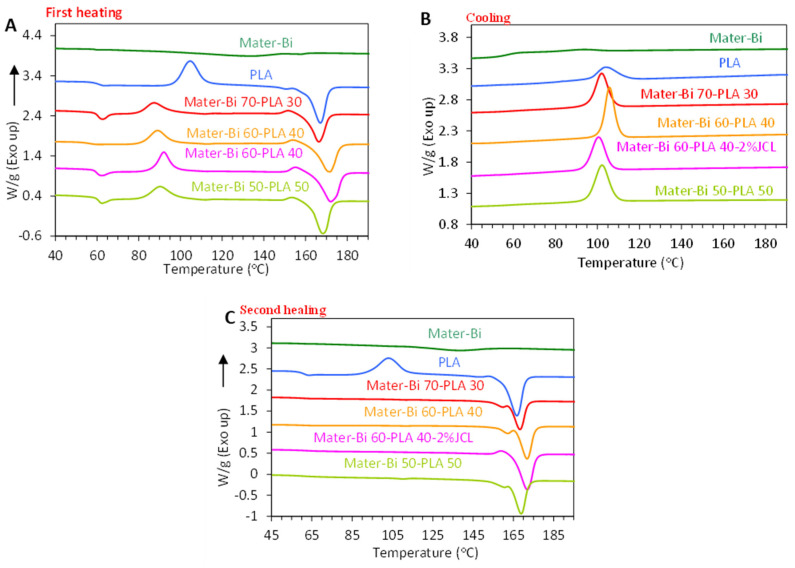
DSC curves of Mater-Bi/PLLA blends during (**A**) first heating scan, (**B**) cooling scan, and (**C**) second heating scan.

Figure 5A–C illustrate the DSC thermograms of different formulations of Mater-Bi/PLLA blends. Results of glass transition (Tg), cold crystallization and melting temperatures (Tcc and Tm, respectively), enthalpy of cold crystallization (Δ*H_cc_*), enthalpy of melting (Δ*H_m_*) and degree of crystallinity (*χ_c_*) from first heating and cooling cycles were set out in Table 4. Mater-Bi^®^ is characterized by a low Tg around −35 °C and a broad melting transition in the range of 115–140 °C with a low magnitude, indicative of a low crystalline degree of Mater-Bi. The low Tg and crystallinity of Mater-Bi account for the high ductility of this polymer blend. On the first heating scan, PLLA displayed a Tg around 62 °C and a melting temperature Tm at 168 °C, and exhibited a cold crystallization peak (TCC) around 106 °C. This cold crystallization peak was maintained during the second heating scan, which indicates a low tendency of PLLA to crystallize during the cooling process [12]. As calculated from the melting enthalpy, the crystalline degree did not exceed 8%, confirming the low crystallinity of PLLA, which is one of the shortcomings of PLLA.

For the Mater-Bi/PLLA blends, the glass transition barely changed regardless of the weight fraction of PLLA present in the blend confirming the immiscibility rule of both biopolymers (Figure 5). At the same time, the cold crystallization temperature in the first heating scan was shifted to lower temperature, with a decrease in Δ*Hcc* by more than 40%, compared with neat PLLA. The cold crystallization completely vanished during the second heating scan, indicating that the presence Mater-Bi promoted the crystallization of PLLA by acting as a nucleating agent. The presence of the coupling agent JCL did not impair the nucleating effect of Mater-Bi, but an increase by about 5 °C in the Tm of PLLA was observed.

**Figure 6:**
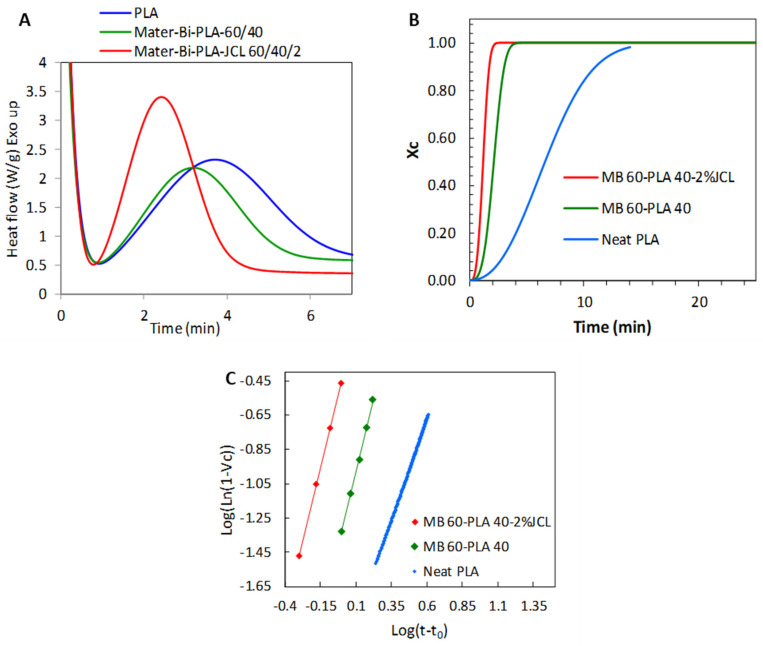
Avrami analysis isothermal data at 120 °C: (**A**) experimental isothermal data, (**B**) the relative crystallinity with time, and the (**C**) Avrami Log–Log plot.

To further demonstrate the nucleating effect of Mater-Bi^®^ on PLLA, the isothermal melt crystallization kinetics at three temperatures were investigated using Avrami’s formalism, from which the relative crystallinity degree X(i) at time t was calculated from the specific heat flow, and Avrami’s index n and rate constant k were extracted by using Equation (5). As shown in Figure 6A, the relative crystallinity vs. time at the different temperatures studied displayed a typical sigmoid shape. From this plot, the crystallization half-time (*t*_1/2_), representing the time needed for 50% of the total crystallization to occur, was extracted and reported in Table 4. *t*_1/2_ of neat PLLA was 6.61 min while it dropped to 2.18 min and 1.23 min for the uncompatibilized and compatibilized blends, respectively. This confirmed that the presence of Mater-Bi^®^ accelerated the crystallization kinetics of PLLA, presumably by acting as a nucleating agent.

Table 5 reports the key parameters of the isothermal crystallization kinetics using the Avrami equation (indicated in Section 2.4) following the procedure developed by Lorenzo et al. [17].

### 3.5. Rheological Properties

**Figure 7:**
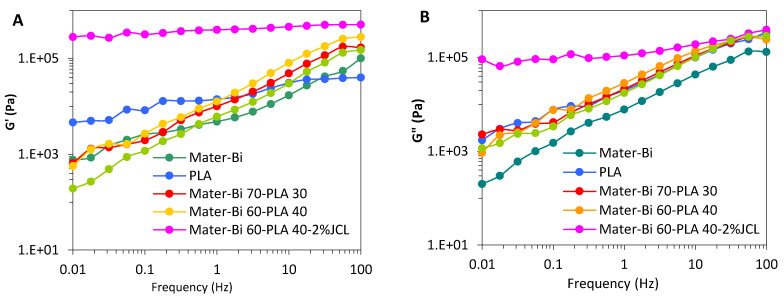

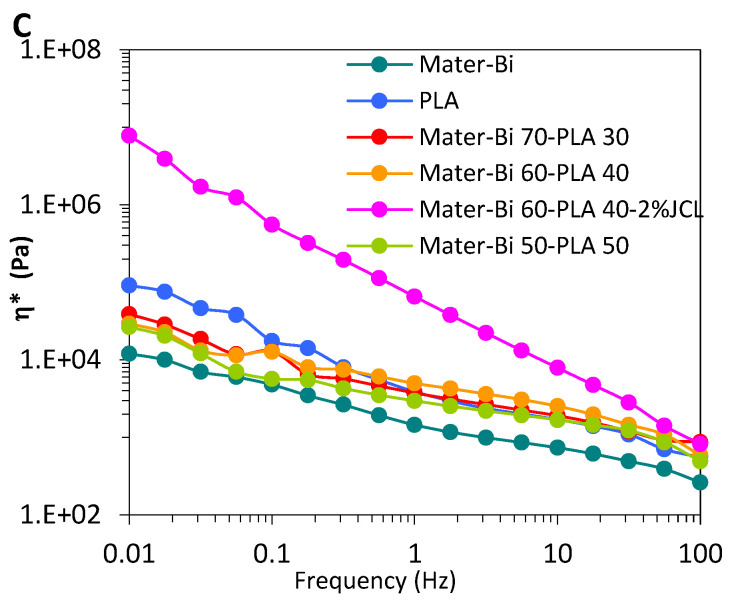
(**A**) Storage modulus (G′), (**B**) loss modulus (G′′), and (**C**) complex viscosity (η*) versus frequency of Mater-Bi/PLLA blends.

The melt rheological behavior of the Mater-Bi/PLLA blends, as well as neat PLLA and Mater-Bi, were investigated by oscillatory sweep measurements of the storage modulus (G′), loss modulus (G′′), and complex viscosity (η*) as a function of frequency (f) in the linear domain (Figure 7) at 160 °C. The Mater-Bi exhibited the lowest melt viscosity at 160 °C with a shear-thinning behavior as the frequency increased, which is typical of viscous melt polymer. In comparison, PLLA demonstrated higher η* at 160 °C. The uncoupled blends follow a shear thinning [28] behavior intermediate between the neat PLA and Mater-Bi. Their viscoelastic character at melt is dominated by a liquid-like behavior with G″ being higher than G′ and the power-low frequency dependence (Figure 7A).

On the other hand, an obvious change in the melt rheology was noted in the Mater-Bi 60-PLLA40-JCL blend processed in the presence of 2% JCL. As shown in Figure 7A,B, G′ experiences a huge upward shift with more than two orders of magnitude at the low frequencies, with G′ and G″ being nearly frequency-independent and G′ being higher than G″ (G′ being two times higher than G″ at the same frequency), which is indicative of a dominance of the elastic over the liquid character. The melt viscosity (Figure 7C) also increased sharply, namely, at lower frequencies (f < 1Hz) with a pronounced shear-thinning behavior but tends to converge to the same level for the uncoupled blend at high frequencies (f ≈ 100 Hz). This obvious increase in the melt elasticity and melt viscosity of the blend processed in the presence of JCL is due to the concomitance of two effects: (i) the enhancement of interfacial interaction and entanglement ability of Mater-Bi^®^ and PLLA chains through the reaction of the epoxy groups of JCL with the hydroxyl and carboxyl groups of PLLA and Mater-Bi^®^. This effect accounts for the improvement in the toughness of the Mater-Bi^®^-PLLA-JCL blend. The second reason would be (ii) the inevitable occurrence of chain extension and coupling in Mater-Bi and PLLA phases, induced by the reaction of the epoxide groups of JCL with carboxyl or hydroxyl chain end groups of polyester [29]. The resulting chain branching and coupling engender an enlargement in the chain relaxation time of the macromolecules, which explains the huge increase in G′ and η* at low frequencies range.

### 3.6. Disintegration Test

**Figure 8:**
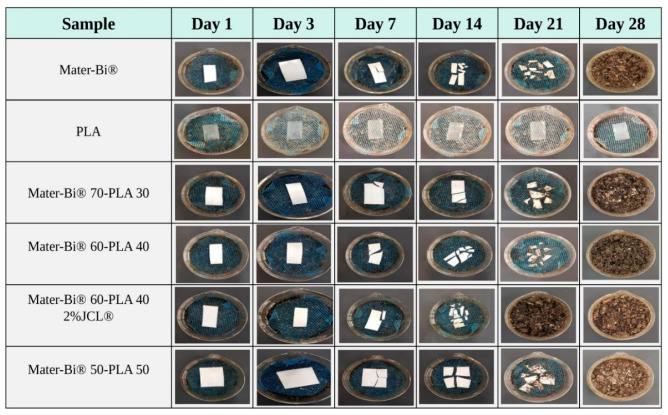
Macroscopic appearance of biodegradation in compost with 50% moisture at 58 °C.

The films from neat Mater-Bi^®^, PLLA, and the Mater-Bi^®^/PLLA blends were buried in controlled compost soil at a temperature of 58 °C and 50% relative humidity. The biodegradation of the films was inspected by their physical appearance as a function of burial time. It was very clear from Figure 8 that Mater-Bi has the highest rate of disintegration compared to neat PLLA. The visual observation of the recovered samples showed that the fragmentation and the disintegration of Mater-Bi^®^ started after a week, unlike PLLA, which exceeded 100 days after the burial. Although both biopolymers exhibit hydrolyzable structure, it seemed that PLLA is less prone to hydrolytic degradation, which is likely the steric shielding effect of methyl side groups of PLLA [30], and to the relatively high Tg of PLLA, making the enzyme less accessible to the polymer as the macromolecular chain are frozen without any possibility for water molecules to diffuse inside the polymer. As for the blends Mater-Bi^®^/PLLA, the embrittlement process was observed on day 7, similar to the neat Mater-Bi^®^. This finding was expected since polymer blends often lead to higher degradation rates explained by the formation of dispersed particles of the minor component in the immiscible system [31]. Moreover, Mater-Bi^®^ is a mixture of starch and polyester, which means blending it with a hydrophobic polyester (PLLA) will increase the hydrophilic nature of the blend and thus facilitating the penetration of the microorganisms into the blends and increasing the degradation rate.

### 3.7. Film Blowing

**Figure 9:**
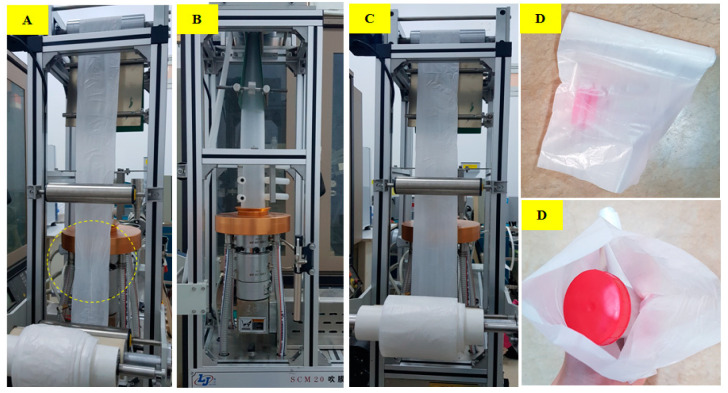
Mater-Bi/PLLA (60/40) blend (**A**), compatibilized Mater-Bi^®^/PLLA (60/40) blend during the film-blowing process (**B**,**C**), and the final product (**D**).

The extrusion-blowing tests were run for Mater-Bi^®^ 60-PLLA 40, with and without the coupling agent, JCL (Figure 9). In the absence of JCL, it was very difficult to produce stable continuous bubbles with the aforementioned formulation. Even so, multiple bubble defects were identified, including wrinkles, bubble breathing, and axisymmetric periodic variation of the bubble diameter during the blowing extrusion. The melt velocity was maintained constant during the extrusion processing, while BUR-DDR parameters were constantly changing due to the instability of the bubble formation. On the other hand, in the presence of 2% JCL, in the blend of Mater-Bi^®^60-PLLA-40, blown-film was easily produced with good reproducibility. The quality of the bubbles was kept constant, without any defect at BUR-DDR values equal to 1.6 and 12.5 respectively, demonstrating the key role of the compatibilizer in the enhancement of film blowing properties of the Mater-Bi^®^/PLLA blends. Similar results were also obtained with a Mater-Bi^®^/PLLA ratio of 70–30 in the presence of 2% JCL. The improvement in the blowing properties in the presence of JCL could be explained by the evolution in the melt rheology of the blend when JCL was added. Indeed, as shown in Section 3.5, an obvious increase in the melt elasticity (G′) and melt viscosity of the blend when JCL was added, which led to a higher melt strength during the blowing process that contributed to stabilizing the bubble and prevent collapsing during the blowing process [31]. The tensile properties of the films were tested along the machine direction (MD) and in the transverse direction (TD). The results are presented in Table 6.

The first interpretation that can be made from the data presented in Table 6 is that the mechanical properties of the blown film were higher in the machine direction (MD) compared to the (TD) due to the orientation of the polymer chains in the process [31]. Moreover, comparing the results in Section 3.1 of the as-extruded films and blown film fabricated from the same formulation, it is noticeable that the elastic modulus, elongation at break, and tensile strength of the material were higher than the as-extruded film, this could be ascribed to the fact that blowing process induces the tensile parameters of the material. Secondly, the tensile properties of the blend, the standard material (HD-PE) usually employed for film blowing in industries, and the blend PBAT/TPS were quite different from the ones found in the case of the Mater-Bi^®^/PLLA blend. Although the elongation at break for the blend used in this study was low compared to the values of the pristine polymer (Mater-Bi^®^) (570%), HD-PE (420%), and the PBAT/TPS blend (620%), the UTS and the Young modulus improved drastically by reaching 49.9 MPa and 2321 MPa, while the values of neat Mater-Bi, HD-PE, and PBAT/TPS blend were 34/20/18 MPa and 510/600/MPa, respectively. In conclusion, the flexibility and the acceptable mechanical properties in both MD and TD of compatibilized Mater-Bi/PLLA blend compared to conventional petroleum-based polymers, neat biopolymers, or classic polymer blends, may offer the possibility of being used as a potential substitute in food packaging applications.

## 4. Conclusions

To enhance the stiffness and strength of Mater-Bi^®^, PLLA was added at a content between 30 to 50% to produce heterogeneous Mater-Bi^®^/PLLA blends. A multifunctionalized epoxide (compatibilizing agent Joncryl-ADR^®^ (JCL) was added to improve the interfacial interaction between the Mater-Bi^®^ and PLLA phases. The inclusion of PLLA onto Mater-Bi^®^ at different contents strongly enhanced the strength and stiffness of the blend, as much higher as the content of PLLA is elevated. However, a decline in the elongation at break was observed, which is expected given the heterophase microstructure of Mater Bi^®^/PLLA blends. The enhancement in the stiffness was further supported by DMA analysis, which depicted the evolution of the stiffness or modulus according to the temperature. The crystallinity kinetics of the PLLA phase was shown to be enhanced by the presence of Mater-Bi by acting as a nucleating agent for PLLA. This further contributed to widening the application windows of Mater-Bi^®^/PLLA to over 50 °C. Interestingly, in the presence of JCL, it was possible to produce thin blown-film with good reproducibility by the extrusion-blowing process. The improvement in the blowing properties in the presence of JCL was explained by the increase in the melt elasticity (G′) and melt viscosity of the blend, which led to a higher melt-strength during the blowing process. The fabricated films were characterized by satisfactory mechanical properties, which can readily be up-scaled for the industrial production of flexible packaging films that are eco-sustainable and compostable under different compositing conditions.

## Figures and Tables

**Table 1:** Characteristics of the polymers.

Material	Information
Mater-Bi^®^ Grade: Mater-Bi^®^ YI0140/C	Supplier: Novamont MFI *: 33.36 g/10 min Density: 1.24 g/cc Melting point: 190 °C
PLLA Grade: Ingeo^®^ biopolymer 3D870	Supplier: Natureworks MFI *: 9 g/10 min Density: 1.24 g/cc Melting point: 130 °C
Joncryl-ADR^®^	Supplier: BASF Epoxy equivalent weight (g/mol) 310 Glass transition temperature:59 °C

MFI * = Melt flow index.

**Table 2:** Blends composition.

Composition.	Mater-Bi^®^ (wt%)	PLLA (wt%)	Joncryl-ADR^®^ (wt%)
Mater-Bi^®^	100	-	-
PLLA	-	100	-
MB/PLLA-70/30	70	30	-
MB/PLLA-60/40	60	40	-
MB/PLLA/JCL-60/40/2	60	40	2
MB/PLLA-50/50	50	50	-

**Table 3:** Blown extrusion parameters.

Blown Extrusion Parameters	Values
Lay-flat width	118 mm
Bubble diameter	75 mm
Die diameter	45 mm
Die gap	0.8 mm
Film thickness	0.04 mm
Blow-up ratio (BUR)	1.6
Draw blow-up ratio (DDR)	12.5

**Table 4:** The main thermal properties of the different formulations of Mater-Bi/PLLA related to the first scan.

	Tg_1_ (°C)	Tg_2_ (°C)	Tcc (°C)	Tm (°C)	Δ*H_cc_* (J/g)	Δ*Hm* (J/g)	*χ_c_* (%)
Mater-Bi	−33	-	-	133	-	-	-
PLLA		62	106.1	168	35.09	−42.21	7.6
Mater-Bi 70-PLLA 30	−38	63.8	88.6	167.3	19.56	−32.95	47.5
Mater-Bi 60-PLLA 40	−37	62.1	90.5	172.6	19.15	−38.8	52.8
Mater-Bi 60-PLLA 40-2%JCL	−40	62.8	93.1	173	21.59	−41.11	52.4
Mater-Bi 50-PLLA 50		63.8	91.6	169.5	20.96	1.66	41.5

**Table 5:** Parameters of the Avrami fit obtained from isothermal analysis at 120 °C of 60/40 Mater-Bi/PLLA blend: Avrami exponent (n), predicated half-time crystallization (*t*_1/2_ Theo), experimental half-time (*t*_1/2_ exp), overall transformation constant (k), and correlation coefficient (R^2^).

Blend	Avrami Index (n)	*t*_1/2_ Theo (min)	*t*_1/2_ Exp (min)	K (min-n)	R^2^
Neat PLLA	2.36	6.613	6.217	0.01512	0.9998
Mater-Bi 60-PLLA 40	3.47	2.185	2.167	0.0457	0.9998
Mater-Bi 60-PLLA 40–2%JCL	3.35	1.231	1.33	0.0812	1

**Table 6:** Main tensile properties of the corresponding blend in the machine direction (MD) and transverse direction (TD) of the blown film, and comparison with film from HDPE and neat Mater-Bi^®^.

	Elongation at Break (%)		Young Modulus (MPa)		Ultimate Tensile Strength (MPa)	
**Mater-Bi^®^**	**570**		**510.2**		24	
HD-PE	420		600		26	
Mater-Bi^®^/60%-PLLA 40 –2%JCL	**MD**	**TS**	**MD**	**TS**	**MD**	**TS**
	113	107	2321	1971.5	49.9	42.9

## Data Availability

Not applicable.

## Supplementary Materials

The following supporting information can be downloaded at: https://www.mdpi.com/article/10.3390/polym15010153/s1, Figure S1: SEM images of the cryogenic fractured surfaces of the Mater-Bi/PLA 70/30 and 50/50 (wt%); Figure S2: Storage Modulus E’ (A), Tan δ (B) versus Temperature of Mater-Bi, and Mater-Bi/PLLA 60/40/2JCL blends; Figure S3: Thermogram DSC of Mater-Bi and Mater-Bi/PLLA in the low temperature domain focusing on the glass-transition temperature.
