# Supplementary material for: Processing Polymer Blends of Mater-Bi® and Poly-L-(Lactic Acid) for Blown Film Application with Enhanced Mechanical Strength
Source: Polymers (Basel). 2022 Dec 29;15(1):153. doi: 10.3390/polym15010153 (PMC9823894; doi:10.3390/polym15010153)
Supplement: Supplementary file 1 [file polymers-15-00153-s001.zip › polymers-2097218-supplementary.pdf]

Supplement Material

Processing Polymer blends of Mater-Bi and poly-L-(latic acid) for blown film application with enhanced mechanical strength.

Table S1 :

	Elongation at break (%)	Young Modulus (Mpa)	Tensile strength (Mpa)	Ultimate sterngth (Mpa)	Yeild strength (Mpa)	Toughness (MJ/m3)
Mater-Bi	161.2	0.5	4.77	10.8	9.6	9.25
PLA	3.4	2.15	58.52		72.16	1.23
Mater-Bi 70-PLA 30	14	0.89	16.4	26.1	23.33	2.14
Mater-Bi 60-PLA 40	10.8	0.766	27.66	36.441	33.35	4.1
Mater-Bi 60-PLA 40-2%JCL	51.3	1.8	27.7	49.37	45.3	16.57
Mater-Bi 50-PLA 50	9.7	1.98	40.8	55.05	54.3	4.04

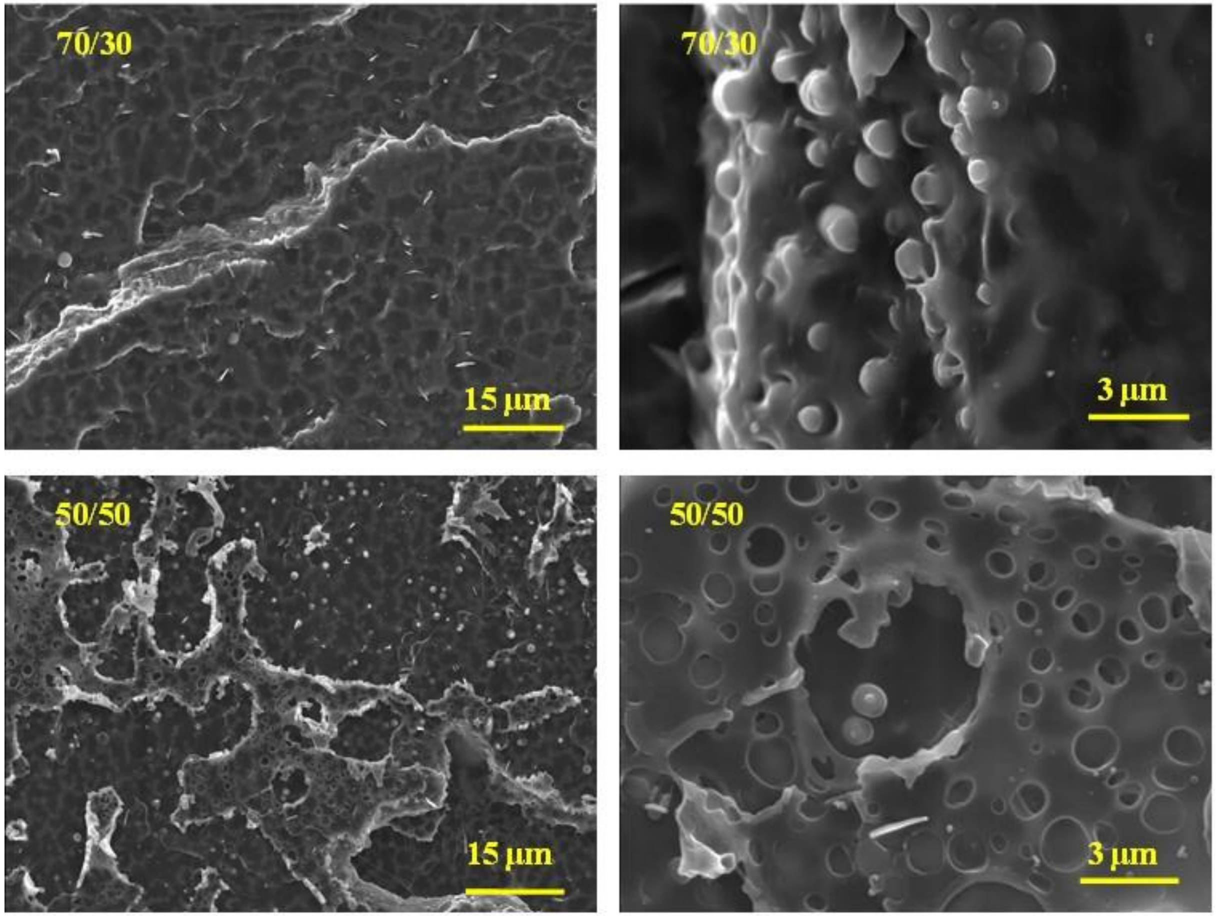

Figure S1: SEM images of the cryogenic fractured surfaces of the Mater-Bi/PLA 70/30 and 50/50 (wt%)

A

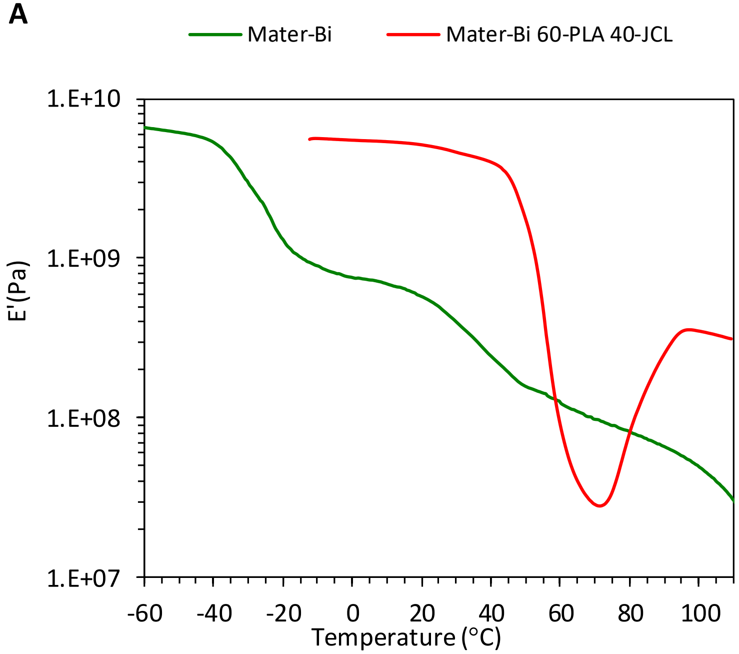
Mater-Bi
Mater-Bi 60-PLA 40-JCL
1.E+10
1.E+09
1.E+08
1.E+07
E'(Pa)
-60
-40
-20
0
20
40
60
80
100
Temperature (°C)

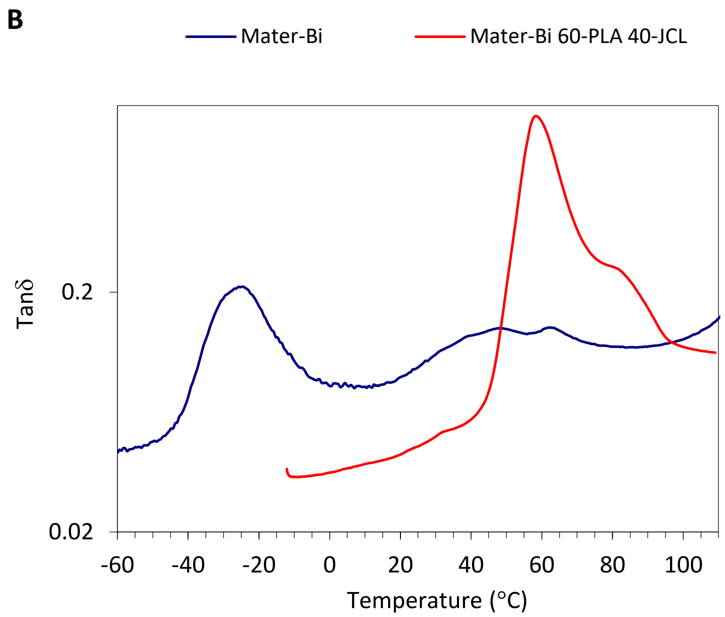

Figure S2: Storage Modulus E′ (A), Tan δ (B) versus Temperature of Mater-Bi, and Mater-Bi/PLLA 60/40/2JCL blends.

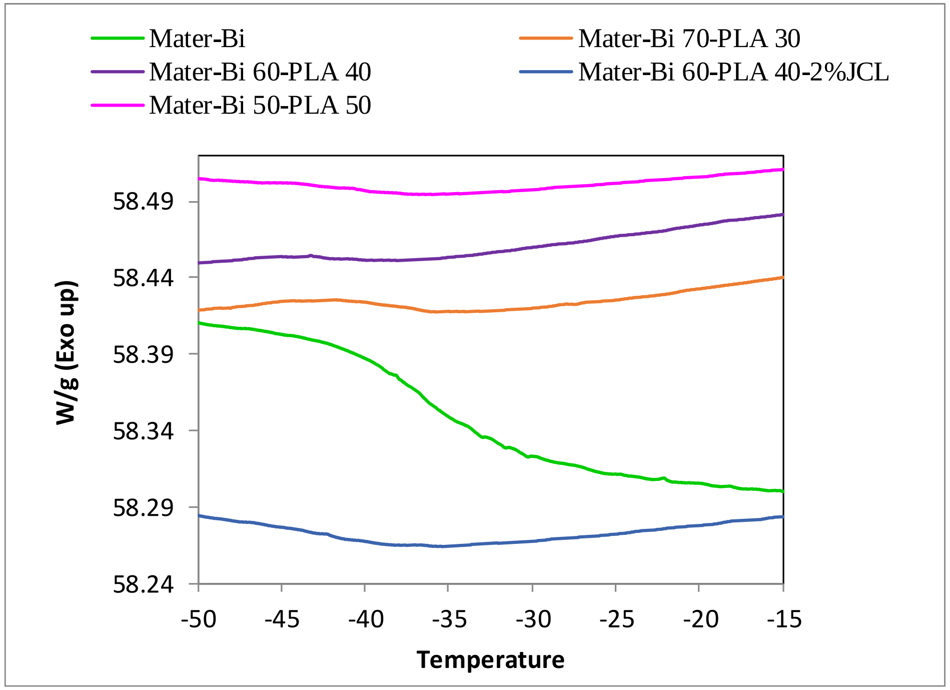

Figure S3: Thermogram DSC of Mater-Bi and Mater-Bi/PLLA in the low temperature domain focusing on the glass-transition temperature
